# A Cadaveric Case Report of Bilateral Accessory Anterior Bellies of the Digastric Muscles

**DOI:** 10.7759/cureus.75938

**Published:** 2024-12-18

**Authors:** Hannah J Grimmett, Kamal A Abouzaid, Ava Greenberg, Niharika Dar, Ketsia Kimbimbi, Hadiseh Faridi Tavana, Ahmad Imam

**Affiliations:** 1 Department of Anatomical Sciences, William Carey University College of Osteopathic Medicine, Hattiesburg, USA

**Keywords:** accessory anterior belly of digastric muscle, anterior belly of the digastric muscle, bilateral accessory bellies of the anterior digastric muscles, cadaveric case report, submandibular triangle, submental triangle

## Abstract

The digastric muscle is a suprahyoid muscle that is composed of an anterior belly and a posterior belly, which originate from the first and second pharyngeal arches, respectively, and they are innervated by the nerves of these arches. The digastric muscles are involved in the elevation of the hyoid bone and depression of the mandible during mastication, speech, and swallowing.

In this report, we present the rare case of bilateral accessory anterior belly of the digastric muscles (ABDMs) that originated from the digastric fossa, medial to the anterior bellies. Both accessory anterior bellies crossed the midline and joined the intermediate tendon on the contralateral side. The crossing of the accessory anterior bellies along with the normal anterior bellies resulted in an M-shaped muscular configuration at the submental region. The innervation was identified bilaterally as a branch from the ipsilateral mylohyoid nerve supplying the normal and accessory anterior bellies.

It has been hypothesized that abnormal migration of neural crest cells, traditionally responsible for the development of the first pharyngeal arch, results in the formation of an accessory ABDM. Although anomalies of the ABDM are rare, they can present a diagnostic dilemma during radiologic and surgical evaluation. Accessory ABDMs can be used as a muscle or tendon graft in surgical reconstructive procedures; aside from this, they are of limited biomechanical significance.

## Introduction

The digastric muscle is a suprahyoid muscle that is composed of two bellies, an anterior belly and a posterior belly [1,2]. Embryologically, the anterior belly originates from the first pharyngeal arch while the posterior belly originates from the second pharyngeal arch [1,2]. The two bellies receive their innervation from two different sources [2]. The anterior belly of the digastric muscle (ABDM) is innervated by the mylohyoid nerve, which arises from the inferior alveolar nerve, a branch of the mandibular division of the trigeminal nerve [3]. In contrast, the posterior belly of the digastric muscle (PBDM) is innervated by the facial nerve [3]. The ABDMs serve as the lateral borders of the submental triangle while both the anterior and posterior bellies form the inferior boundaries of the digastric triangle, and the mandible forms the base for both the submental and digastric triangles [3,4].

The ABDM arises from the digastric fossa on the mandible and descends laterally toward the hyoid bone; the PBDM originates from the mastoid process and passes anteriorly toward the hyoid bone [3]. The anterior and posterior bellies join each other at the intermediate tendon, which is restrained by a fibrous sling that attaches to the hyoid bone [1,3]. The digastric muscle’s main function is to elevate the hyoid bone and depress the mandible; this action contributes to speech, stabilization while swallowing, and chewing [5].

Anatomical variations of the digastric muscle have primarily been observed in cadavers; these variations can be both unilateral and bilateral [4]. It has been proposed that abnormal migration of neural crest cells, traditionally responsible for the development of the first pharyngeal arch, results in the formation of the accessory ABDM [6]. Yamada (1935) and Zlabek (1933), as cited by Anderson and Tucker, proposed a classification system for anatomical deviations of the ABDM. In this classification system, the possible variations of the accessory ABDM dictate the type of deviation from the norm: atavistic (classical) type, origin (anterior) type, insertion (posterior) type, mixed type, complex (composite) type, and deletion type. The origin (anterior) type, which can present unilaterally or bilaterally, can be further divided into the continuous origin type and the muscle-bundle origin type. If the anterior accessory belly overlaps the ABDM, then it is considered a continuous origin type; however, if the anterior accessory belly is separated from the ABDM, then it is considered a muscle-bundle origin type [7].

In this report, we identify a rare case of bilateral accessory ABDMs that join the contralateral intermediate tendons and retain the ipsilateral innervation. Our findings contribute to the limited literature discussing this rare class of digastric muscle variation and its potential functional and clinical implications.

## Case presentation

During routine dissection of the anterior cervical region in an adult formalin-fixed female cadaver who died at the age of 74, we observed an interesting muscular anomaly in the submental region that appeared as a “McDonald's sign.” The cadaveric donor was a Caucasian who was obtained from the University of Southern Alabama Anatomical Gift Program with Parkinsonism as the documented cause of death.

**Figure 1:**
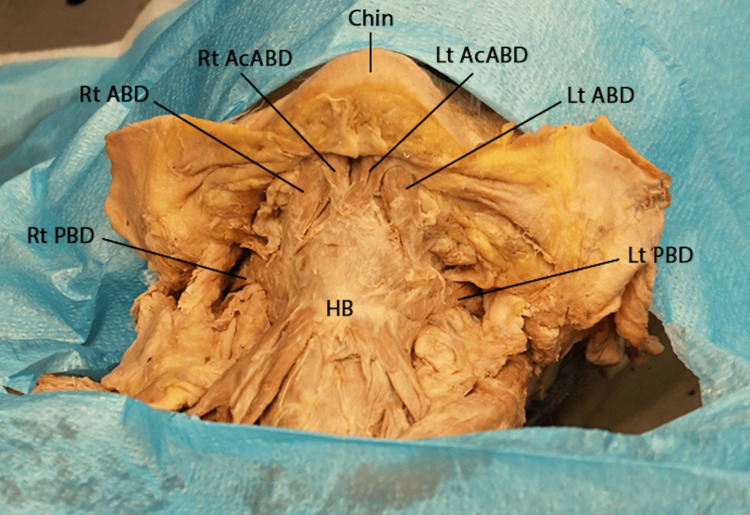
Anterior view of the neck showing the submental and submandibular triangles Lt AcABD: left accessory anterior belly of digastric; Lt ABD: left anterior belly of digastric; Lt PBD: left posterior belly of digastric; Rt AcABD: right accessory anterior belly of digastric; Rt ABD: right anterior belly of digastric; Rt PBD: right posterior belly of digastric; HB: hyoid bone

**Figure 2:**
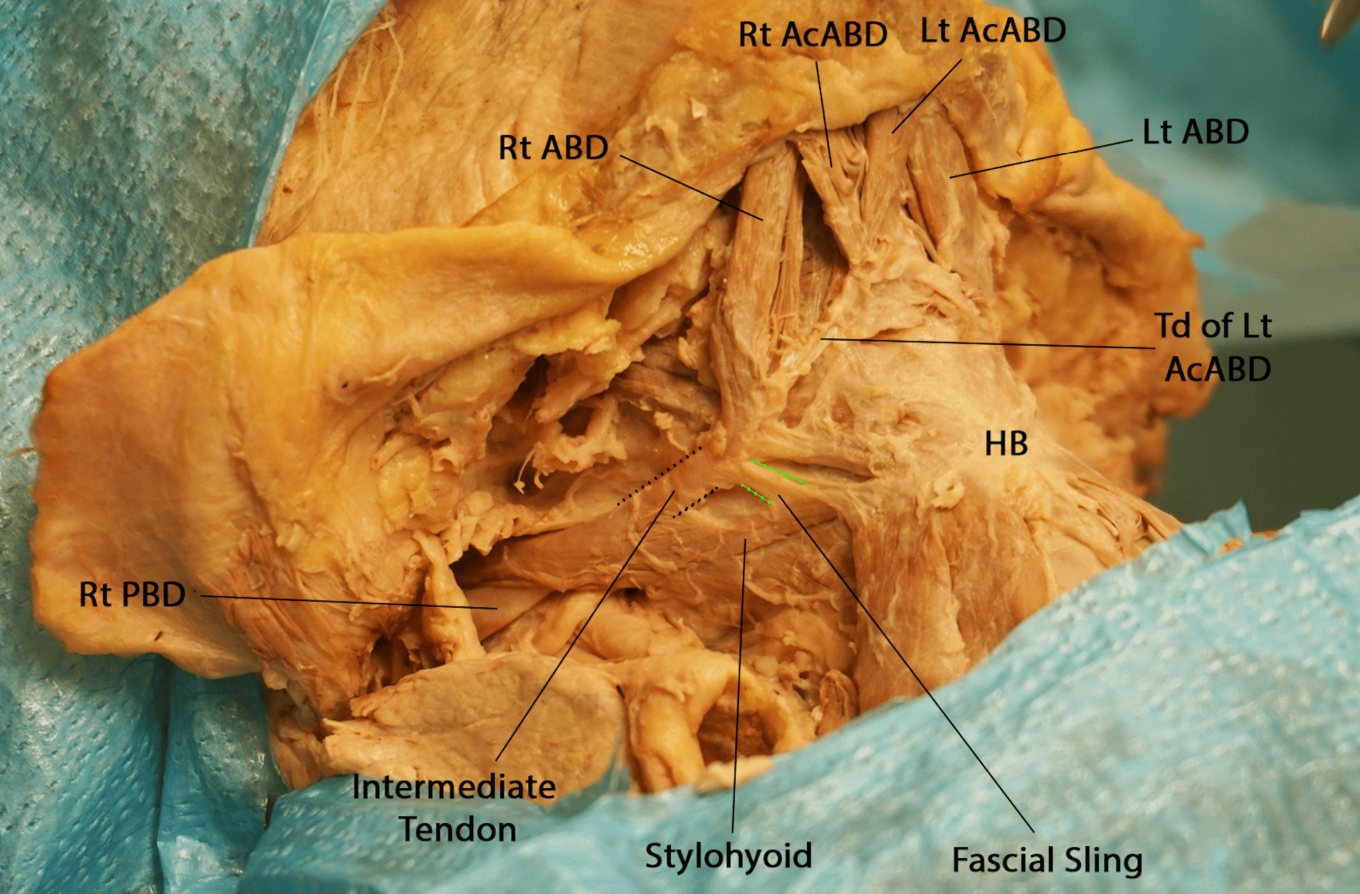
Right anterolateral view of the upper neck region showing the intermediate tendon of the digastric muscle and the facial sling Lt AcABD: left accessory anterior belly of digastric; Td of Lt AcABD: tendon of the left accessory anterior belly of digastric; Lt ABD: left anterior belly of digastric; Rt AcABD: right accessory anterior belly of digastric; Rt ABD: right anterior belly of digastric; Rt PBD: right posterior belly of digastric; HB: hyoid bone

During the dissection assignment of the anterior triangle of the neck, the platysma was dissected, bilaterally, and the muscle flaps were retracted superiorly. The investing layer of the deep cervical fascia was removed to expose the subdivisions of the anterior cervical triangle and their contents. The anterior and posterior bellies of the digastric muscles were dissected bilaterally to show the boundaries of the submental and digastric triangles. On each side, the anterior belly was observed arising from the digastric fossa on the inner aspect of the mandible, and the posterior belly was attached to the medial side of the mastoid tip. The two bellies were joined by an intermediate tendon that descended close to the hyoid bone (Figures 1, 2). A sling of deep cervical fascia connected the intermediate tendon to the hyoid bone (Figure 2).

**Figure 3:**
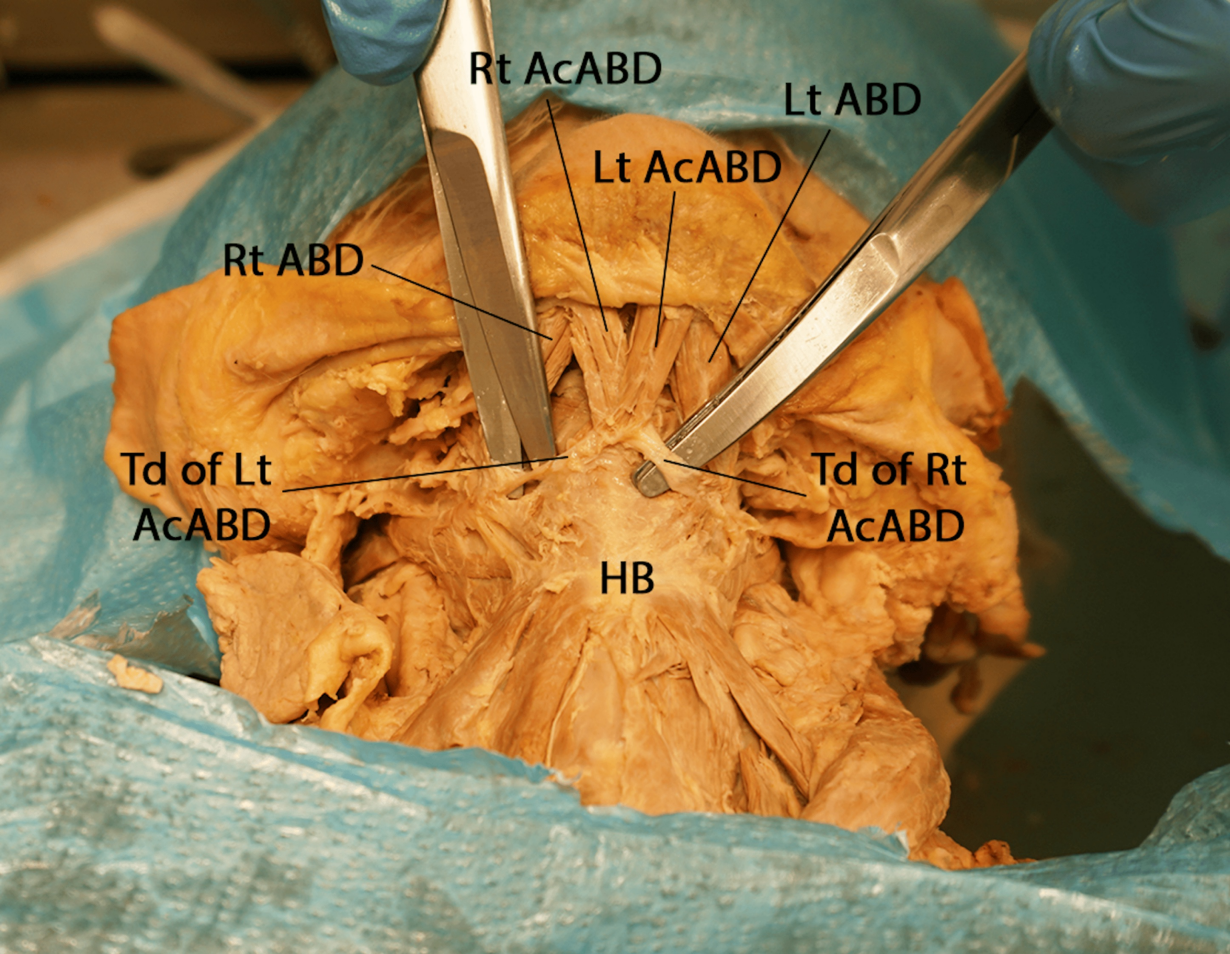
Anterior view of the submental region showing the accessory anterior bellies of the anterior digastric muscles and their tendons Lt AcABD: left accessory anterior belly of digastric; Lt ABD: left anterior belly of digastric; Td of Lt AcABD: tendon of the left accessory anterior belly of digastric; Rt AcABD: right accessory anterior belly of digastric; Rt ABD: right anterior belly of digastric; Td of Rt AcABD: tendon of the right accessory anterior belly of digastric; HB: hyoid bone

Moreover, we observed bilateral accessory anterior bellies originating from the digastric fossa, medial to the anterior bellies (Figure 3).

**Figure 4:**
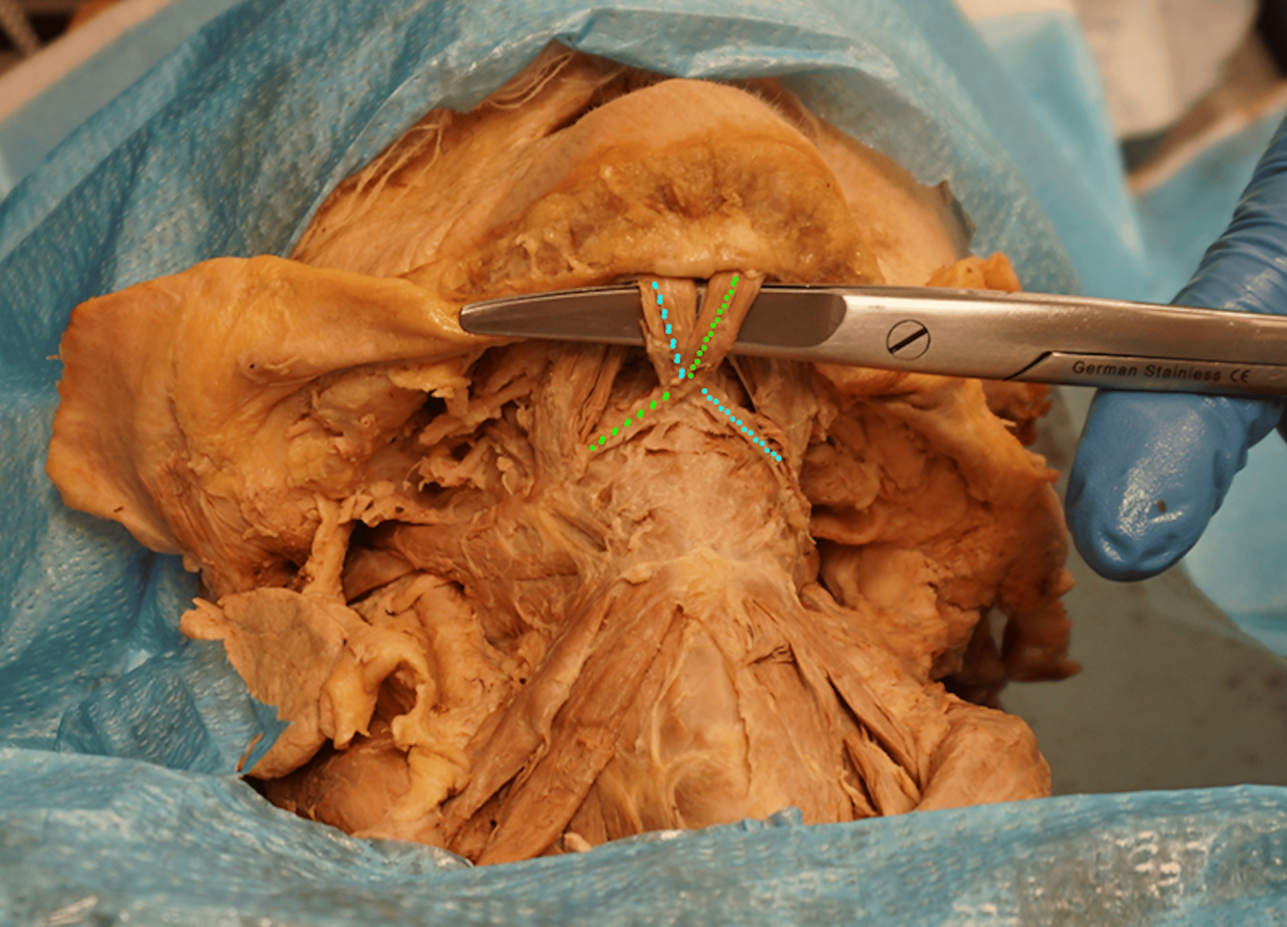
The crossing of the accessory anterior bellies of the anterior digastric muscles is delineated with the dotted lines

Both accessory anterior bellies cross to the contralateral side and join the contralateral intermediate tendon (Figure 4).

**Figure 5:**
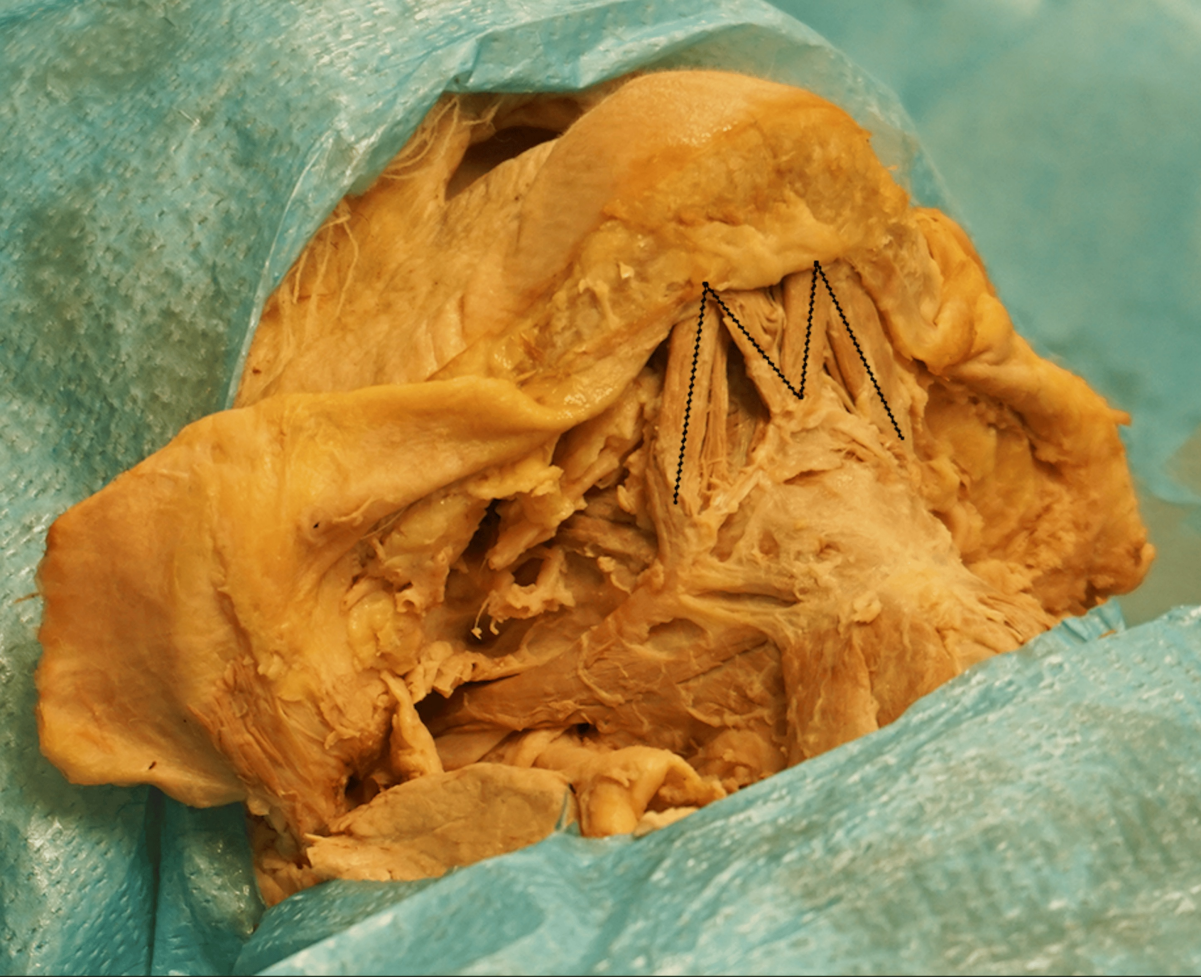
The M-formation formed by the normal anterior bellies and the crossed accessory anterior bellies of the digastric muscles

The crossing of the accessory anterior bellies along with the normal anterior bellies resulted in the M-shaped muscular configuration of the submental region (Figure 5). The innervation was identified bilaterally as a branch from the ipsilateral mylohyoid nerve supplying the normal and accessory anterior bellies.

## Discussion

Kim and Loukas conducted a systematic review of variations of the accessory ABDM; they found the anomaly to be more prevalent in the Asian population. In fact, both unilateral and bilateral variations were identified in 51.7% (707/1,368) of cadavers including the Asian population, and only 31.4% (325/1,036) of cadavers excluding the Asian population. Additionally, out of 326 cadavers, accessory ABDMs were more commonly bilateral than unilateral with the percentages being 53.7% and 46.3%, respectively [2]. Regarding the prevalence of bilateral accessory ABDM insertion into the intermediate tendon, De-Ary-Pires et al. identified the anomaly in 2.7% (4/146) of cadavers; however, there are no details provided to determine if the insertion of these cases is into the ipsilateral or contralateral intermediate tendon [8]. Given this, a bilateral accessory ABDM originating from the digastric fossa and inserting into the contralateral intermediate tendon in a Caucasian female is rare.

While the digastric muscle is not generally considered a muscle of mastication, it has been proven to have some involvement with chewing [9]. Both the anterior and posterior bellies of the digastric muscle contract, simultaneously, in depression and retrusion of the mandible [10]. Mastication involves the participation of both the anterior and posterior bellies of the digastric muscle; however, peak muscle activity differs between them. The posterior belly showed two peak activity points (major and minor) corresponding with minimal peak activity and maximal peak activity of the anterior belly; the maximal peak activity of the anterior belly correlated with the minor activity peak of the posterior belly [9].

Both the ABDM and PBDM participate in the elevation of the hyoid bone when swallowing; both bellies follow a pattern of crescendo-decrescendo activity with peak activity times ranging from slightly earlier than the midway point between activation and termination of the muscle activity. Munro compared the activity of the digastric muscle during the swallowing of water and saliva. The swallowing of saliva had a longer duration of muscle involvement when compared to swallowing water [9].

Unfortunately, if the presence of an accessory ABDM is unknown, it can be misdiagnosed as a pseudomass on the mylohyoid muscle; it is differentiated from a tumor on CT via the lack of abnormal contrast enhancement. Due to the close relationship between the ABDM and the mylohyoid muscle, it would be vital to know the existence of an accessory anterior belly prior to the surgical removal of a mass involving the mylohyoid muscle [11]. In our case, the accessory ABDM crosses the submental triangle, so it is possible for it to be mistaken for an enlarged lymph node via CT or MRI; the knowledge of the accessory belly’s existence will decrease the probability of incorrect diagnoses and, potentially, additional tests.

There are also specific, beneficial surgical implications associated with having an accessory ABDM. Permanent damage to the marginal mandibular branch of the facial nerve (MMBFN) results in the loss of innervation to the ipsilateral depressor anguli oris and depressor labii inferioris resulting in an asymmetrical smile; these circumstances arise either due to inadvertent injury or intentional sacrifice of the nerve for tumor clearance during head and neck surgery [12]. While there are procedures to correct the damaged nerve, most result in additional weakness of the lower lip [13]. In patients where MMBFN loss is in isolation or part of total facial nerve paralysis, the ABDM can be used for reconstruction in a paralyzed lower lip and has very successful outcomes [12]. Possessing an accessory ABDM would be even more beneficial for patients suffering from MMBFN paralysis, as the ABDM could be left intact while the accessory belly is used for the reconstruction of the paralyzed lower lip. This abnormality seen in this report can be classified as a muscle-bundle origin (anterior) type anatomical deviation. In this variant, the accessory muscle and its tendon cross to the contralateral side, which provides more length of the muscle and tendon fibers, and hence, one could speculate that it could be used in a more distant facial muscle reconstruction.

Although the accessory ABDM is innervated by the ipsilateral mylohyoid nerve, by joining the contralateral intermediate tendon, it acts on the contralateral side of the hyoid bone and mandible. For instance, if there was an injury to the mylohyoid nerve on the right side, one should expect to see a negative effect in moving the mandible on the same side as the nerve injury, since the anterior belly of the digastric is innervated by the ipsilateral nerve [3]. The crossing of the accessory anterior bellies has been previously reported; however, to our knowledge, no studies have explored the potential functional benefit of the accessory ABDM in cases of nerve damage on the opposite side. A noteworthy observation in the current case is that the accessory belly functions on the contralateral side while being innervated by the ipsilateral nerve. Therefore, we hypothesize that if the contralateral nerve is injured, leading to paralysis of the contralateral anterior digastric, the accessory ABDM on the ipsilateral side may compensate, owing to its ipsilateral innervation and contralateral functional role.

## Conclusions

Although anomalies of the anterior belly of the digastric muscle are rare, knowledge of them is essential during surgical dissection of the neck. Moreover, they can be mistaken for a lymph node or a mass during radiological examinations and may result in unnecessary excisional biopsy. This report photographically documented the exciting findings of the current case and will contribute to the limited literature on anomalies in the digastric muscle. Moreover, it will help physicians understand this specific variant to be able to provide optimal patient care.
